# Quality Improvement in Apple Ciders during Simultaneous Co-Fermentation through Triple Mixed-Cultures of *Saccharomyces cerevisiae*, *Pichia kudriavzevii*, and *Lactiplantibacillus plantarum*

**DOI:** 10.3390/foods12030655

**Published:** 2023-02-02

**Authors:** Lujun Hu, Xiaodie Chen, Rui Lin, Teng Xu, Dake Xiong, Li Li, Zhifeng Zhao

**Affiliations:** 1 College of Biological Engineering, Sichuan University of Science and Engineering, Yibin 644005, China; 2 Liquor Brewing Biotechnology and Application Key Laboratory of Sichuan Province, Sichuan University of Science and Engineering, Yibin 644005, China; 3 College of Biomass Science and Engineering, Sichuan University, Chengdu 610000, China

**Keywords:** cider, mixed-culture fermentation, non-*Saccharomyces* yeast, *Lactiplantibacillus plantarum*, antioxidant property

## Abstract

This study explored the effect of the combination of *Saccharomyces* yeast, non-*Saccharomyces* yeast (*Pichia kudriavzevii*), and *Lactiplantibacillus plantarum* during cider fermentation on physicochemical properties, antioxidant activities, flavor and aroma compounds, as well as sensory qualities. Ciders fermented with the triple mixed-cultures of these three species showed lower acid and alcohol content than those fermented with the single-culture of *S. cerevisiae*. The antioxidant activities were enhanced by the triple mixed-culture fermentation, giving a higher 1,1-diphenyl-2-picrylhydrazyl (DPPH) radical scavenging rate and total antioxidant capacity; specifically, the SPL5 cider showed the highest DPPH radical scavenging rate (77.28%), while the SPL2 gave the highest total antioxidant capacity (39.57 mmol/L). Additionally, the triple mixed-culture fermentation resulted in improved flavor and aroma with a lower acidity (L-malic acid) and higher aroma compounds (Esters), when compared with the single-culture fermented ciders (*Saccharomyces cerevisiae*); more specifically, the SPL4 cider resulted in the highest total flavor and aroma compounds. In addition, sensory evaluation demonstrated that ciders produced using the triple mixed-cultures gained higher scores than those fermented using the single-culture of *S. cerevisiae*, giving better floral aroma, fruity flavor, and overall acceptability. Therefore, our results indicated that the triple mixed-cultures (*S. cerevisiae*, *P. kudriavzevii*, and *L. plantarum*) were found to make up some enological shortages of the single *S. cerevisiae* fermented cider. This study is believed to provide a potential strategy to enhance cider quality and further give a reference for new industrial development protocols for cider fermentation that have better sensory qualities with higher antioxidant properties.

## 1. Introduction

Apples are rich in nutrients such as sugar, vitamins, dietary fiber, and trace minerals as well as polyphenolic bioactive compounds, giving them the ability to lower blood lipids and prevent many types of cancers [1,2]. Apple cider is one of the directions of apple processing, which preserves its original nutrients and provides a unique aroma, ultimately meeting the demand of functional-food consumers. In China, Yanyuan Fuji apple from Yanyuan County of Sichuan Province has been proved to be an excellent raw material for the production of functional foods with improved nutritional values [3]. Yanyuan Fuji apples have unique characteristics such as being pollution free with varieties of flavor substances and higher concentrations of sugar and polyphenols. However, little research has been conducted on the cider fermentation with Yanyuan Fuji apples.

Cider is a fermented apple product, possessing the properties of fruit wines and according to the Chinese alcoholic beverage industry’s policy, it is a product of low alcohol content and low grain consumption. It is well-known that apple cider fermentation is a complex biochemical process, including the conversion of sugars into alcohols with the production of aroma compounds through cider-related microbial communities, which result in the complexity and diversification of the product [4]. It has been extensively known that the apple cider fermentation process includes alcoholic fermentation (AF) and malolactic fermentation (MLF); these fermentations affect the qualities of the apple cider [5]. During apple cider fermentation, yeasts have an essential impact on the production of aroma compounds by releasing some volatile compounds from the apples, simultaneously synthesizing new volatile substances [6]. However, for a long time, the production of apple cider has been practiced using yeasts, especially the commercial *Saccharamyces cerevisiae*, which results in the production of an insufficient and monotonous aroma in the product and cannot characterize the unique flavor of the apple thoroughly [7]. Consequently, it is vital to explore the suitable microbial strains as the starter culture for the fermentation of apple ciders.

Nowadays, non-*Saccharomyces* yeasts, including species of *Pichia* [8], *Hanseniaspora* [9], *Zygosaccharomyces* [10], *Schizosaccharomyces* [11], *Wickerhamomyces* [12], *Torulaspora* [13], and *Williopsis* [14], receive more and more attention due to their desired enological characteristics as the potential adjuncts to *S. cerevisiae*; such inclusions form high concentrations of flavor compounds. The varieties of enzymes from these multiple strains can interact with apple precursors and enhance the complexity of apple cider [9,15]. However, most of these non-*Saccharomyces* yeasts cannot accomplish AF alone. Because of this reason, the employment of non-*Saccharomyces* yeast in association with *S. cerevisiae* provides a viable solution to the problem. In addition, the combination of non-*Saccharomyces* yeast with *S. cerevisiae* has been found to improve the ester formation during AF [16].

Immediately after the alcoholic fermentation, lactic acid bacteria (LABs), which exist naturally in the fermentation mix or are added later, start to transform L-malic acid to L-lactic acid (Malolactic fermentation, MLF); this results in a higher flavor complexity of the product [6,17,18]. *Oenococcus oeni* is generally adapted as a starter culture to conduct MLF [5]. The employment of *Lactiplantibacillus* spp. when conducting MLF, however, has attracted a lot of attention because they can produce and secrete more flavor-related enzymes than *O. oeni.* The genus *Lactiplantibacillus* is a significant one amongst LABs. More recently, some studies reported that *Lactiplantibacillus* spp. was able to evoke MLF as favorably as *O. oeni* when co-cultured with *S. cerevisiae* and non-*Saccharomyces* yeast [19,20]. In addition, many studies showed that some *Lactiplantibacillus* strains could be used as a potential adjunct to *Saccharomyces* yeast to enhance fruit wine quality [21,22]. Moreover, apple juice contains good substrate materials that can be fermented using the probiotic *L. plantarum* [23]. Dimitrovski and coworkers showed that the apple juice fermented using *L. plantarum* possessed an improved sensory quality, such as better taste, increasing the acceptability of the product [24]. Etxeberria and coworkers showed that *L. plantarum* can de-esterify, de-methylate, de-sugar, and convert the bound phenols into free phenols, making them more easily available for digestion and absorption by the body [25].

Continuous improvement in the apple cider quality is essential for the cider industry’s growth. Microbiological aspects, such as the choice of yeasts and LABs, are the vital factor for cider quality improvements. Yeasts can improve the sensory scores of apple cider by producing metabolites, which enhance its color, aroma, and structure. *Saccharomyces* and non-*Saccharomyces* yeasts used as the mixed starter cultures are becoming increasingly interesting for improving the quality and the complexity of the fruit wine. Yu and coworkers found that the mixed-cultures of *S. cerevisiae* with *Schizosaccharomyces pombe* significantly improved the aroma and taste quality of ciders [11]. In addition, *L. plantarum* fermentation was found to enhance the antioxidant activities of apple ciders. Li and coworkers applied *S. cerevisiae* and *L. plantarum* as the mixed starter cultures to improve the antioxidant activity of the fermented apple cider [26]. However, to the best of our knowledge, reports of comprehensive research on the influence of triple mixed-cultures of *Saccharomyces* yeasts, non-*Saccharomyces* yeasts, and LABs during the fermentation of apple ciders have not yet been published.

Based on the above literature, the apple cider quality was studied with simultaneous co-cultures of different strains of *S. cerevisiae*, *P. kudriavzevii*, and *L. plantarum* for the fermentation of apple cider. Some crucial parameters were determined in the study, including basic cider parameters, antioxidant activities, and aroma compounds. The study was expected to provide valuable information about the triple mixed-culture fermentation of *Saccharomyces* yeasts, non-*Saccharomyces* yeasts, and LABs on the chemical and sensory properties of apple ciders, which would help enologists to optimize the starter cultures so as to further improve apple cider quality.

## 2. Materials and Methods

### 2.1. Yeast and Bacterial Strains and Culture Media

Sixteen microbial strains were employed in the study, including the commercial strain of *S. cerevisiae* Angel (Angel Yeast Co., Ltd., Yichang, China), *Saccharomyces cerevisiae* (SCFF203, SCFF205, SCFF211, SCFF215, and SCFF233), *Pichia kudriavzevii* (SCFF163, SCFF185, SCFF204, SCFF207, and SCFF214), and *Lactiplantibacillus plantarum* (SCFF19, SCFF107, SCFF169, SCFF180, and SCFF200). The yeast strains were cultured on yeast extract-peptone-dextrose (YPD) medium at 28 °C. All the strains of *L. plantarum* were grown on de Man–Rogosa–Sharpe (MRS) medium at 37 °C. The yeasts and bacteria employed in this study came from the Culture Collection of Food Microorganisms of Sichuan University of Science and Engineering (Yibin, China).

### 2.2. Apple Cider Fermentation

Apple ciders were made according to the previous studies [26,27] with some modifications. The apples were washed and then drained. Subsequently, after removing the seeds, apples were cut into small pieces and crushed with a food-grade juicer to obtain an apple juice. Ascorbic acid (0.08%) was then added to the apple juice to prevent the enzymatic browning; this apple juice was then pasteurized at 95 °C for 5 min in a conical flask and subsequently cooled down to room temperature. Before inoculation, all of the yeast strains of *S. cerevisiae* and *P. kudriavzevii* were cultured in YPD liquid medium via shaking at 150 rpm in a shaker at 28 °C for 24 h, while all *L. plantarum* strains were cultured in MRS liquid medium at 37 °C for 16 h. *S. cerevisiae*, *P. kudriavzevii*, and *L. plantarum* were incubated under anaerobic conditions for two generations to make these microbial strains grow in an anaerobic environment. Afterward, cells were separated from the liquid medium with centrifugation at 4500× *g* for 10 min. The pellets were washed with sterile saline (0.85%) and centrifuged at 4500× *g* for 10 min, and the process was repeated three times successively. The cells were then resuspended in the pasteurized apple juice for subsequent fermentation. All microbial strains were inoculated at a final count of 10^7^ CFU/mL in 1000 mL apple juice. Six fermentation tests were subsequently carried out: (1) single inoculation with the commercial strain of *S. cerevisiae* Angel (SCA); (2) simultaneous inoculation with *S. cerevisiae* SCFF205, *P. kudriavzevii* SCFF185, and *L. plantarum* SCFF200 (SPL1); (3) simultaneous inoculation with *S. cerevisiae* SCFF233, *P. kudriavzevii* SCFF163, and *L. plantarum* SCFF107 (SPL2); (4) simultaneous inoculation with *S. cerevisiae* SCFF203, *P. kudriavzevii* SCFF214, and *L. plantarum* SCFF19 (SPL3); (5) simultaneous inoculation with *S. cerevisiae* SCFF211, *P. kudriavzevii* SCFF207, and *L. plantarum* SCFF180 (SPL4); and (6) simultaneous inoculation with *S. cerevisiae* SCFF215, *P. kudriavzevii* SCFF204, and *L. plantarum* SCFF 169 (SPL5). The pasteurized apple juice with no inoculation was considered the control (AJ). The simultaneous inoculation method was used in this study with the inoculum ratio of *S. cerevisiae*, *P. kudriavzevii*, and *L. plantarum* at 2:2:1 [8,9]. The apple juice fermentation was carried out at 20 °C in the dark for 16 days. At the end of the fermentation process, the apple cider was collected from the strains and lees by centrifugation at 7000× *g* for 10 min (at 4 °C). The supernatant was collected and stored at −20 °C to prevent the interferences from oxygen and light, which was subjected to further analysis.

### 2.3. Physicochemical Analysis

Physicochemical properties of the samples were analyzed according to the method described by the previous report [13] with some modifications. The pH value was determined using a pH meter (PH-100, Lichen, Shanghai, China). The soluble solid content (SSC) was measured by a digital refractometer (RA-620, Kyoto, Japan). The total acidity content was determined with acid-base titration with 0.1 M NaOH, while the reducing sugar content was determined by 3,5-dinitrosalicylic acid based on the method in GB/T 15038–2006. The alcoholic content of apple ciders was determined on the basis of the second method in GB 5009.225–2016.

### 2.4. Determination of Antioxidant Activity

The antioxidant activities of the apple juice and ciders were determined with 1,1-diphenyl-2-picrylhydrazyl (DPPH) free radical superoxide anion reducing power and the total antioxidant activity, which were calculated according to the method described by the previous report [28] with some modifications.

### 2.5. GC-TOF-MS Analysis

GC-TOF-MS analysis was carried out with an Agilent 7890 gas chromatograph and a time-of-flight mass spectrometer (MS) on the basis of the previously described method [27] with some modifications. The Agilent DB-5MS capillary column was used in the system with helium as the carrier gas. The injection volume was one μL in splitless mode. The front inlet purge flow was 3 mL/min, and the gas flow rate through the column was 1 mL/min. The initial temperature was 50 °C (Holding for 1 min), subsequently raised to 310 °C at 10 °C/min and maintained at this temperature for 8 min. The injection, transfer line, and ion source temperatures were 280 °C, 280 °C, and 250 °C, respectively. Electron ionization (Electron impact mode at 70 eV) spectra in the *m/z* range from 50 to 500 were acquired in full-scan mode at 12.5 spectra per second after a solvent delay of 6.25 min. The compounds were determined using semi-quantitative analysis on the basis of the added internal standard (2-octanol).

### 2.6. LC-MS/MS Analysis

LC-MS/MS analyses were performed according to the method described by Zhou and coworkers with some modifications [29] using the UHPLC system (Vanquish, Thermo Fisher Scientific), equipped with a Waters ACQUITY UPLC BEH Amide column (2.1 mm × 100 mm, 1.7 μm) together with Q Exactive HFX mass spectrometer (Orbitrap MS, Thermo Fisher Scientific). Two solvents were used to elute: mobile phase A (25 mmol/L ammonium acetate and 25 mmol/L ammonia hydroxide in water) and mobile phase B (100% acetonitrile). The auto-sampler temperature was kept at 4 °C, and a 2 μL aliquot of samples was injected. The QE HFX MS was employed to collect MS/MS spectra in an information-dependent acquisition mode through the acquisition software (Xcalibur, Thermo). In this mode, the acquisition software continuously evaluates the full scan MS spectrum. The operating conditions of the electrospray ionization source were applied as follows: sheath gas flow rate was 30 Arb; aux gas flow rate was 25 Arb; the capillary temperature was 350 °C; full MS resolution was 120,000; MS/MS resolution was 7500; collision energy was 10/30/60 eV in NCE mode; and spray voltage was 3.6 kV (Positive) or −3.2 kV (Negative), respectively.

### 2.7. Sensory Analysis

Sensory analysis was performed on the basis of the previous method with some modifications [30]. The sensory properties of the finished ciders were evaluated by a group of 15 panelists, comprising students and teachers with relevant experiences and background knowledge. The age and gender of the volunteers were not taken into consideration. Cider quality was evaluated through six attributes: fruity taste, sweetness, bitterness, sourness, flavor, and overall acceptability; these attributes were scored according to the nine-point hedonic scale (one indicated poor, and nine represented excellent). About 50 mL of each cider sample was served in a wine glass, labeled with a random code number; then, evaluation was conducted under white light and at room temperature. The sensory quality of each finished cider was assessed by calculating and plotting the average scores of all characters.

### 2.8. Statistical Analysis

Each experiment was conducted in triplicate, and the result was expressed as means ± standard deviation (SD). The difference between experimental groups was analyzed with Duncan’s multiple comparison test using IBM SPSS version 26 (SPSS Inc., Chicago, IL, USA), and the level of statistical significance was accepted to at least 5%. Hierarchical cluster analysis (HCA) and principal component analysis (PCA) were performed with Origin 9.0 (Hampton, MA, USA).

## 3. Results and Discussion

### 3.1. Changes in Physicochemical Parameters

Physicochemical characteristics of apple juice and ciders (mono-fermented with *S. cerevisiae* or co-fermented with *S. cerevisiae*, *P. kudriavzevii*, and *L. plantarum*) are displayed in Table 1. At the end of the fermentation, the pH values of the apple ciders were lower than that of the apple juice, except for SPL3 cider. The total acid contents of the apple ciders ranged from 3.62 mg/mL to 5.32 mg/mL, which were higher than that of the apple juice, confirming the previous result [8]. The cider fermented with *S. cerevisiae* Angel (SCA) showed the highest acidity content (5.32 ± 0.12 mg/mL), which was higher than that of other finished apple ciders of simultaneous fermentation with *S. cerevisiae*, *P. kudriavzevii*, and *L. plantarum*, indicating that triple mixed-culture fermentations had a biological deacidification ability.

The sugar consumption abilities varied among all simultaneous fermentation processes with *S. cerevisiae*, *P. kudriavzevii*, and *L. plantarum*. The reducing sugar contents of the apple ciders fermented with simultaneous fermentation with *S. cerevisiae*, *P. kudriavzevii*, and *L. plantarum* changed in an opposite direction; for example, the reducing sugar contents for SPL2 (5.07 ± 0.01 mg/mL), SPL4 (4.55 ± 0.01 mg/mL), and SPL5 (6.07 ± 0.03 mg/mL) changed more quickly than that for SCA (mono-fermentation with *S. cerevisiae* Angel)(7.00 ± 0.01 mg/mL), while that for SPL1 (15.89 ± 0.06 mg/mL) and SPL3 (25.44 ± 0.07 mg/mL) showed a slower decrease. The alcohol percentage in all apple ciders fermented with simultaneous co-fermentation processes with *S. cerevisiae*, *P. kudriavzevii*, and *L. plantarum* were lower than that in the mono-fermented cider with *S. cerevisiae* Angel (SCA), revealing that the simultaneous co-fermentation process had a relatively lower alcohol-producing capacity.

### 3.2. Comparative Analysis of Antioxidant Activity

**Figure 1:**
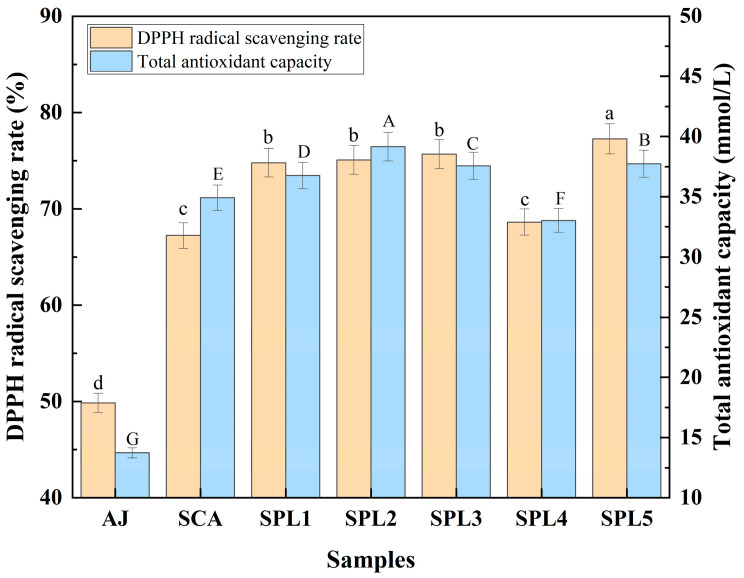
Changes in 1,1-diphenyl-2-picrylhydrazyl (DPPH) radical scavenging rate and total antioxidant activity of apple ciders after mono- and co-fermentation. Data with different letters (a, b, c, d) in DPPH radical scavenging rate and (A, B, C, D, E, F, G) in total antioxidant activity are significantly different (*p* < 0.05), respectively.

Total antioxidant activity and DPPH free radical superoxide anion reducing power in apple juice and finished apple ciders were studied. Overall, the antioxidant activities of apple ciders were higher than that of apple juice. Figure 1 showed that the total antioxidant activity and DPPH free radical scavenging rate of apple ciders after mono- and co-fermentation were higher than those in apple juice before fermentation. Moreover, total antioxidant activity and DPPH free radical scavenging rate in ciders of triple mixed-cultures (for example, SPL1, SPL2, SPL3, and SPL5) were significantly higher than those in the single-culture (SCA). Specially, SPL5 cider showed the highest DPPH radical scavenging rate (77.28% ± 0.12%), while SPL2 cider showed the highest total antioxidant capacity (39.57 ± 0.06 mmol/L), suggesting that the triple mixed-cultures improved the antioxidant activities of the apple cider. Our results were consistent with the previous studies [26,31].

### 3.3. Analysis of Esters, Higher Alcohols, Aldehydes, and Ketones

In this study, a total of 31 compounds, including 21 esters, 5 aldehydes, 3 higher alcohols, and 2 ketones, were determined by GC-MS in apple juice and the finished ciders (Table 2). The total response values of the triple mixed-culture fermented apple ciders (SPL) were significantly higher (*p* < 0.05) than those in the single *S. cerevisiae* fermented cider (SCA), indicating that the triple mixed-cultures improved the production of aroma-producing substances because a mixed-culture fermentation of yeasts and bacteria contributed these compounds to regulate the wine aroma complexity [32]. SPL4 ciders had the highest total compound concentrations (3105.6 ± 356.62 μg/L), which were significantly higher than those in other fermented ciders.

The most abundant compounds were esters in the finished ciders (Table 2). Esters are compounds that are considered special in having an essential impact on cider flavor, which provide pivotal qualities concerning desired fruity aromas [33,34]. A total of 21 ester compounds were determined, including 7 ethyl esters, 5 methyl esters, and 9 other esters (Table 2). Ester concentrations in AJ and SCA samples had no significant difference (*p* > 0.05); however, those in SPL1, SPL2, SPL3, SPL4, and SPL5 apple ciders were significantly higher (*p* > 0.05), suggesting that the mixed cultures helped the production of more esters, which was consistent with previous studies [30,34,35]. In particular, SPL1 cider was characterized by the highest level of esters (2348.14 ± 355.68 μg/L). The concentrations of ethyl esters in triple mixed-culture fermented ciders were significantly higher than those in the mono-fermented cider. This was especially true for ethyl tetradecanoate, ethyl isovalerate, and ethyl phenylacetate.

**Figure 2:**
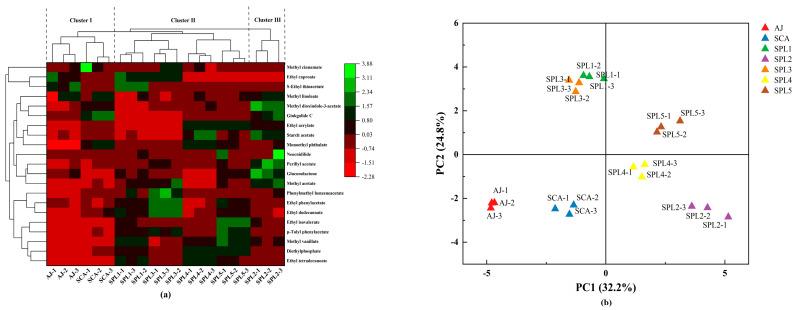
Compounds determined by GC-MS in apple juice and ciders. (**a**) Cluster heatmap of easter compounds in apple juice and ciders. (**b**) Principal component analysis (PCA) based on esters, higher alcohols, aldehydes, and ketones of apple juice and apple ciders.

The heatmap cluster analysis was used to analyze the differences of ester compounds among different apple ciders (Figure 2a). The results displayed the increase or decrease in ester compound formation in each triple mixed-culture fermentation compared with the apple juice and control cider (SCA). Moreover, the ester compounds in the apple juice and ciders were divided into three clusters. Ester components in AJ and SCA cider were grouped in cluster I, while components in SPL1, SPL3, SPL4, and SPL5 ciders were grouped in cluster II, while SPL2 cider was alone grouped in cluster III. These results showed that the ester compound profiles were different not only between the single-culture and mixed-culture fermentations but also among different mixed-culture fermentations with different strains.

A small number of aldehydes, higher alcohols, and ketones were also identified and quantified in the finished ciders. Higher alcohols (which were deemed to be one of the most significant precursors of esters) were conducive to fresh fruity notes and were believed to give a pleasant attribute to the aromatic complexity of fruit wines when their concentrations were below 300 mg/L [27,36]. In this study, the contents of higher alcohols in apple ciders fermented with mixed-cultures were higher (from 39.12 ± 0.47 μg/L to 79.53 ± 7.65 μg/L) than those in the initial apple juice (6.67 ± 0.44 μg/L) and single-culture fermented cider (34.37 ± 2.51 μg/L). Moreover, alcohols, together with organic acids, contribute to the production of esters with a pleasant taste. Aldehydes are generally believed to contribute off-flavors to the apple ciders [37]. Our observations showed that the triple mixed-culture fermentation formed significantly lower aldehyde contents than single *S. cerevisiae* fermentation. The production of ketones was increased considerably in all the ciders with the triple mixed-culture fermentation, compared to the cider with pure fermentation (SCA). Consequently, ciders fermented with mixed microbial strains not only increased esters but also enhanced higher alcohols, aldehydes, and ketones.

For further differentiation of the compounds in apple juice, single-culture fermented cider, and co-culture fermented ciders, PCA analysis of the GC-MS data was conducted. The first and second components accounted for 32.2% (PC1) and 24.8% (PC2) of the total variation, respectively. The scatter plot displayed that apple juice and cider samples were separated from each other (Figure 2b). Apple juice (AJ) and the control cider (SCA) were positioned in the third quadrant. Ciders produced by the mixed-culture fermentation were in the first quadrant (SPL5), second quadrant (SPL1 and SPL3), and fourth quadrant (SPL2 and SPL4).

### 3.4. Analysis of Organic Acids, Polyphenols, and Terpenoids

The starter culture has an essential effect on organic acids, polyphenols, and terpenoids produced in the apple ciders. A total of 86 compounds were determined by LC-MS/MS in all samples, including 44 organic acids, 34 polyphenols, and 8 terpenoids (Table 3).

**Figure 3:**
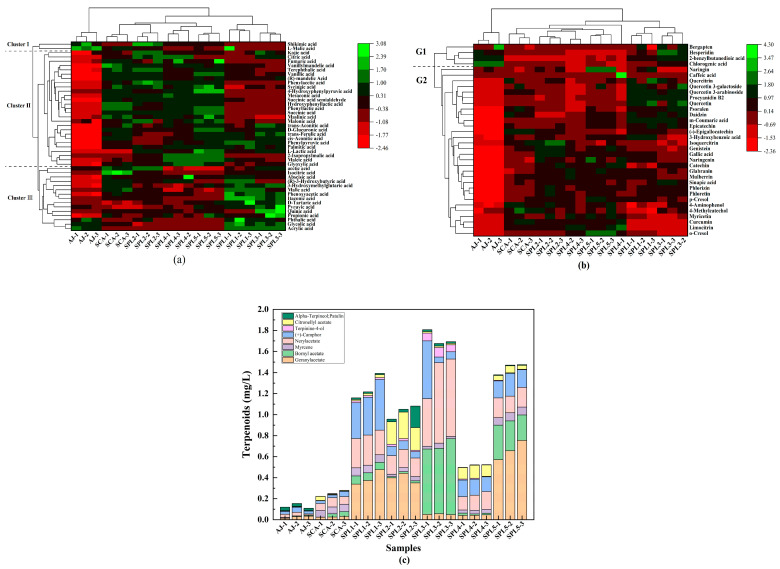
Compounds determined by LC-MS/MS in apple juice and ciders fermented using single-culture and triple mixed-cultures. Organic acids (**a**), polyphenols (**b**), and terpenoids (**c**).

Since organic acids in ciders can make a difference in sensory quality and exert an effect on flavor balance by regulating pH, stability, and comprehensive quality of the fruit wine, it is essential to determine organic acid contents [38,39]. The results showed that total organic acids in all the finished ciders ranged from 44.58 ± 1.33 μg/L to 77.77 ± 1.30 μg/L (Table 3). A heatmap analysis showed that those organic acids in apple juice and ciders were mainly assigned into three clusters (Figure 3a). Components in cluster I included shikimic acid and L-malic acid; the level of L-malic acid in the triple mixed-culture fermented ciders was lower than that in apple juice (AJ) and the mono-fermented cider (SCA). Cluster II included 27 organic acids with lower levels of lactic acid in the triple-mixed-culture fermented ciders, while cluster III had 15 organic acids with higher levels of pyruvic acid in the triple mixed-culture fermented ciders. In particular, high contents of malic acid leads to a harsh taste and unpleasant flavor of apple ciders. As displayed in Table 3, lower concentrations of malic acid and higher concentrations of lactic acids were found in the triple mixed-culture fermented ciders with *L. plantarum* (SPL) than those in AJ and the single-culture fermented cider with *S. cerevisiae* (SCA), illustrating that *L. plantarum* might have the ability of deacidification. Consequently, the results in this study were consistent with the research that *L. plantarum* had the capability of biological deacidification to transform malic acid to lactic acids. Moreover, high concentrations of pyruvic acid and lactic acid had a positive effect on the color stability and the soft perception of fruit wines, respectively [9,40].

Polyphenols had a significant effect on both cider quality and health-promoting properties [41,42]. Previous studies revealed that apple ciders fermented with the cultures of non-*Saccharomyces* yeast and *L. plantarum* were rich in polyphenol contents and had higher antioxidant activities [26]. Among 86 detected compounds in this study, 34 polyphenols were identified (Table 3). Moreover, polyphenol concentrations in AJ and SCA samples were lower than those in the triple mixed-culture fermented ciders; among them, SPL4 cider had the highest level of polyphenols (20.98 ± 5.24 μg/L). According to the heatmap cluster analysis results in Figure 3b, there were significant differences in polyphenol components of ciders fermented using different starters. Moreover, bergapten, hesperidin, 2-benzylbutanedioic acid, and chlorogenic acid clustered into one group (G1). Cluster G2 included 30 phenol compounds; among them, caffeic acid, epicatechin, gallic acid, naringenin, glabranin, phlorizin, and phloretin were found in higher levels in the triple mixed-culture fermented ciders than in the AJ and SCA samples. Hence, the triple mixed-cultures enhanced the formation of many polyphenol compounds, improving the comprehensive quality of apple ciders.

The triple mixed-cultures had significant effects on terpenoids in apple ciders (Table 3, Figure 3c). The addition of *P. kudriavzevii* and *L. plantarum* significantly increased the total amount of terpenoids in apple ciders (from 514.96 ± 11.86 μg/L to 1661.52 ± 48.05 μg/L), especially in SPL3 cider (1661.52 ± 48.05 μg/L), which was about 7-fold higher than that determined in the cider from the single-culture of *S. cerevisiae* (SCA) (231.69 ± 15.56 μg/L). The difference was principally owing to the increase in geranylacetate, nerylacetate, and citronellyl acetate.

### 3.5. Sensory Evaluation

**Figure 4:**
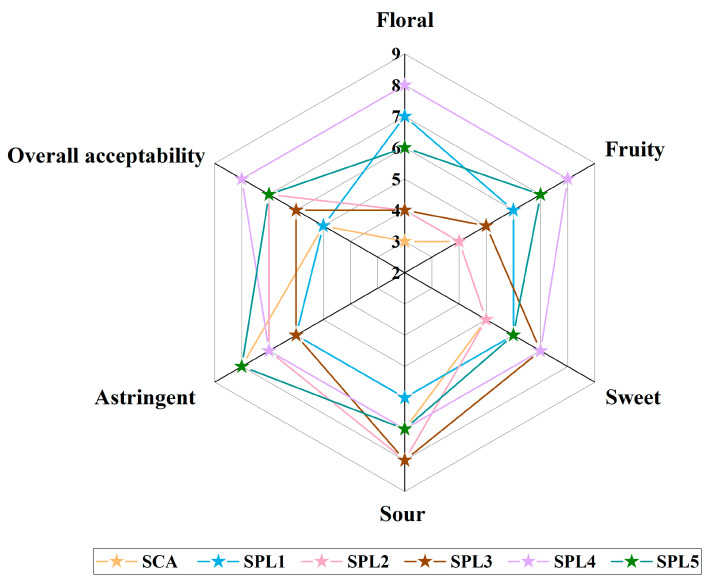
Sensory profiles obtained for ciders fermented with single-culture and triple mixed-cultures.

The sensory evaluation of apple ciders is displayed in Figure 4. Compared to the single commercial yeast fermentation, the sensory scores of ciders with the triple mixed-cultures were much higher. More importantly, the floral aroma, fruity and sweet flavors, and overall acceptability in the ciders of the triple mixed-cultures were significantly improved. The results in this study were consistent with the previous reports, revealing that double mixed-cultures of *P. kudriavzevii* with *S. cerevisiae*, and *L. plantarum* with *S. cerevisiae* had a positive effect on the floral aroma, fruity flavors, and overall acceptability of ciders [8,31,43]. Moreover, sensory evaluation displayed that there were also significant differences among the apple ciders fermented with triple mixed-cultures with different microbial strains, demonstrating that strains were also an essential factor influencing the sensory quality of ciders, which was consistent with the previous study [5].

## 4. Conclusions

In summary, this study investigated the effects of the triple mixed-cultures of *S. cerevisiae*, *P. kudriavzevii*, and *L. plantarum* on the cider quality, including basic physicochemical parameters, antioxidant activities, aroma and flavor compounds, and sensory qualities. The results showed that simultaneous co-inoculations of *S. cerevisiae* together with non-*Saccharomyces* yeast and *L. plantarum* provided a practical way to enhance apple cider quality. Co-inoculating these three species improved antioxidant abilities, compared to those generated by just a single-culture of *S. cerevisiae.* Moreover, the ciders fermented with co-inoculation of the three cultures resulted in higher concentrations of esters, terpenes, and higher alcohols, while exhibiting prominent floral and fruity tastes with a higher overall acceptability. Hence, the results collectively indicated that the triple mixed-cultures provided a potential method to make up the enological shortage of the single-culture fermented cider and further enhanced the quality of apple ciders, facilitating the application of co-culture fermentation technology with different species in cider-making. Our results contributed to the investigation of suitable microbial combinations for cider-making. However, the interactions between different yeasts and *L. plantarum* during fermentation are complex, and the number of these microbial cultures at the end of fermentation therefore needs further study.

## Figures and Tables

**Table 1:** Physicochemical parameters determined in the apple juice before inoculation (Day 0) and apple ciders at the end of fermentation (Day 16).

Parameters Measured	AJ (Day 0)	Treatment (*n* = 3) (Day 16)					
		SCA	SPL1	SPL2	SPL3	SPL4	SPL5
pH	3.79 ± 0.01 b	3.58 ± 0.00 e	3.73 ± 0.01 c	3.54 ± 0.01 f	3.85 ± 0.01 a	3.41 ± 0.01 g	3.68 ± 0.01 d
Total acid (mg/mL)	1.15 ± 0.01 e	5.32 ± 0.12 a	4.54 ± 0.04 b	3.96 ± 0.04 c	5.25 ± 0.07 a	4.17 ± 0.04 c	3.62 ± 0.39 d
SSC (°Brix)	20.00 ± 0.00 a	5.00 ± 0.00 e	6.33 ± 0.29 c	5.00 ± 0.00 e	8.00 ± 0.00 b	5.50 ± 0.00 d	5.00 ± 0.00 e
Reducing sugar (mg/mL)	107.56 ± 0.11 a	7.00 ± 0.01 d	15.89 ± 0.06 c	5.07 ± 0.01 f	25.44 ± 0.07 b	4.55 ± 0.01 g	6.07 ± 0.03 e
Alcohol (% *v*/*v*)	-	11.13 ± 0.12 a	7.97 ± 0.15 d	9.50 ± 0.20 c	7.30 ± 0.20 e	10.23 ± 0.15 b	10.17 ± 0.29 b

Note: Data with different letters (a, b, c, d, e, f, g) in the same row are significantly different (*p* < 0.05). SSC = soluble solid content (expressed as °Brix); “-” means not detected.

**Table 2:** Compounds determined by GC-MS in apple juice and ciders produced by single-culture and triple mixed-culture fermentations.

Number	Compounds	AJ	Treatment (*n* = 3) (μg/L)					
			SCA	SPL1	SPL2	SPL3	SPL4	SPL5
	Esters							
1	Ethyl tetradecanoate	504.85 ± 36.94 c	681.41 ± 41.70 c	1641.06 ± 286.23 a	1036.07 ± 44.70 b	1030.20 ± 61.92 b	1659.25 ± 342.15 a	1238.20 ± 95.31 b
2	Methyl acetate	4.75 ± 0.34 d	6.34 ± 0.59 d	10.42 ± 1.11 bc	16.09 ± 0.33 a	13.55 ± 4.81 ab	6.21 ± 0.29 d	9.87 ± 1.02 c
3	Diethylphosphate	30.85 ± 2.95 g	64.42 ± 0.26 f	121.39 ± 6.89 c	83.00 ± 1.11 d	71.69 ± 1.96 e	189.09 ± 2.93 a	171.63 ± 4.01 b
4	Monoethyl phthalate	-	12.86 ± 0.31 d	33.98 ± 2.76 b	24.35 ± 4.78 c	13.48 ± 1.01 d	45.4 ± 3.22 a	27.32 ± 3.94 c
5	Methyl vanillate	134.79 ± 10.98 d	131.44 ± 1.55 d	294.64 ± 32.28 a	233.73 ± 12.20 b	176.62 ± 10.94 c	242.22 ± 6.05 b	316.94 ± 16.23 a
6	Ethyl dodecanoate	11.01 ± 0.96 e	37.99 ± 2.44 c	57.94 ± 8.3 b	29.14 ± 5.56 d	87.73 ± 2.32 a	29.19 ± 4.96 d	56.26 ± 3.52 b
7	Ginkgolide C	3.29 ± 0.68 bc	33.29 ± 1.3 a	-	33.18 ± 11.79 a	-	10.13 ± 2.45 b	11.11 ± 1.59 b
8	Gluconolactone	122.05 ± 16.39 cde	83.35 ± 3.42 e	159.26 ± 9.38 bc	296.11 ± 49.55 a	130.9 ± 5.95 cd	93.46 ± 3.11 de	169.95 ± 16.58 b
9	Methyl dioxindole-3-acetate	0.78 ± 0.05 a	-	-	9.41 ± 0.01 a	-	-	-
10	S-ethyl thioacetate	3.89 ± 0.74 b	-	23.86 ± 0.05 a	-	-	-	-
11	p-tolyl phenylacetate	1.08 ± 0.05 b	-	-	-	-	-	8.32 ± 1 a
12	Ethyl isovalerate	0.16 ± 0.02 c	0.18 ± 0.01 c	0.75 ± 0.01 c	4.88 ± 0.27 b	1.96 ± 0.31 bc	9.83 ± 0.89 a	8.26 ± 0.23 a
13	Methyl cinnamate	0.04 ± 0.01 c	0.02 ± 0.00 c	0.64 ± 0.10 a	0.35 ± 0.01 b	0.35 ± 0.02 b	0.25 ± 0.01 bc	0.06 ± 0.00 c
14	Neocnidilide	0.01 ± 0.00 d	0.06 ± 0.01 d	0.64 ± 0.06 c	4.05 ± 0.03 a	0.39 ± 0.04 cd	0.06 ± 0.01 d	2.06 ± 0.02 b
15	Ethyl caproate	1.18 ± 0.02 bc	0.34 ± 0.03 c	-	-	2.34 ± 2.03 b	4.27 ± 0.12 a	2.38 ± 0.03 b
16	Methyl linoleate	0.43 ± 0.11 b	0.11 ± 0.01 b	0.47 ± 0.01 b	2.12 ± 1.84 a	3.13 ± 0.18 a	0.72 ± 0.06 b	0.73 ± 0.06 b
17	Ethyl phenylacetate	0.03 ± 0.01 c	-	2.5 ± 0.34 b	2.46 ± 0.46 b	2.38 ± 0.58 b	4.80 ± 0.44 a	0.36 ± 0.03 c
18	Ethyl acrylate	-	1.47 ± 0.30 a	-	-	0.37 ± 0.05 b	-	-
19	Phenylmethyl benzeneacetate	0.49 ± 0.18 b	-	-	0.59 ± 1.02 b	2.16 ± 1.91 a	-	-
20	Perillyl acetate	0.04 ± 0.01 b	-	-	4.93 ± 0.72 a	1.36 ± 0.36 b	-	-
21	Starch acetate	1.32 ± 0.27 a	-	0.60 ± 0.04 a	-	-	1.24 ± 0.04 a	-
	Ʃ_(Sum)_	817.75 ± 14.48 d	1053.27 ± 67.43 d	2348.14 ± 355.68 a	1780.47 ± 92.68 bc	1538.62 ± 71.77 c	2296.11 ± 331.32 a	2023.44 ± 134.23 ab
	Higher alcohols							
1	Glycerol	3.74 ± 0.70 f	26.39 ± 3.47 e	46.46 ± 4.37 b	34.49 ± 2.65 cd	27.8 ± 0.61 de	70.71 ± 8.16 a	39.90 ± 0.82 bc
2	Isoeugenitol	1.97 ± 0.84 d	3.85 ± 0.99 d	7.50 ± 0.37 bc	9.72 ± 2.08 b	10.29 ± 0.76 b	4.68 ± 0.48 cd	26.55 ± 4.47 a
3	3-ethyl-1,2-benzenediol	0.96 ± 0.31 b	4.14 ± 0.36 a	1.11 ± 0.15 b	4.21 ± 0.45 a	1.03 ± 0.56 b	4.15 ± 0.58 a	4.01 ± 0.95 a
	Ʃ_(Sum)_	6.67 ± 0.44 e	34.37 ± 2.51 d	55.08 ± 3.87 c	48.41 ± 4.09 c	39.12 ± 0.47 d	79.53 ± 7.65 a	70.46 ± 3.23 b
	Aldehydes							
1	Vanillin	7.34 ± 2.44 e	85.22 ± 4.20 bc	55.44 ± 4.35 d	91.69 ± 2.24 b	45.97 ± 1.73 d	143.06 ± 10.38 a	77.99 ± 9.88 c
2	Pyridoxal	5.07 ± 0.79 f	562.62 ± 32.57 a	183.20 ± 5.03 d	354.90 ± 10.34 c	113.92 ± 3.44 e	449.05 ± 35.59 b	591.80 ± 7.91 a
3	4-isopropylbenzaldehyde	16.05 ± 4.84 c	23.11 ± 8.81 bc	25.81 ± 6.38 bc	45.74 ± 2.66 a	27.13 ± 0.26 bc	24.88 ± 4.77 bc	33.66 ± 12.48 b
4	2-carboxybenzaldehyde	13.79 ± 3.52 c	11.69 ± 4.27 c	24.24 ± 3.96 ab	31.28 ± 9.19 a	17.03 ± 0.83 bc	23.22 ± 2.37 ab	14.38 ± 2.28 c
5	Benzaldehyde	44.27 ± 12.09 c	31.31 ± 4.94 d	32.90 ± 1.87 d	140.75 ± 4.47 a	38.73 ± 5.01 cd	69.69 ± 1.38 b	48.09 ± 2.9 c
	Ʃ_(Sum)_	86.52 ± 10.88 f	713.95 ± 31.64 b	321.60 ± 4.63 d	664.36 ± 16.63 c	242.79 ± 9.37 e	709.89 ± 38.67 b	765.92 ± 32.97 a
	Ketones							
1	1-(4-hydroxyphenyl)propan-1-one	6.83 ± 1.10 b	6.09 ±4.36 b	6.81 ±0.52 b	12.50 ± 3.68 ab	15.45 ± 8.67 a	7.62 ± 0.61 b	10.75 ± 3.01 ab
2	1,3-diphenyl-2-propen-1-one; Chalcone	0.68 ± 1.18 c	13.56 ± 3.16 b	13.91 ± 0.42 b	26.18 ± 2.55 a	3.63 ± 2.35 c	12.44 ± 2.31 b	27.68 ± 3.59 a
	Ʃ_(Sum)_	7.51 ± 0.59 c	19.65 ±7 b	20.72 ± 0.82 b	38.69 ± 4.74 a	19.08 ± 7.54 b	20.06 ± 1.82 b	38.44 ± 5.84 a
	Ʃ_(Sum: GC-MS)_	921.75 ± 28.57 e	1821.25 ± 19.62 d	2745.53 ± 327.04 bc	2531.93 ± 115.23 c	1839.6 ± 57.72 d	3105.6 ± 356.62 a	2898.26 ± 150.49 ab

Note: “-” means not detected. Data with different letters (a, b, c, d, e, f, g) within each row are significantly different (*p* < 0.05).

**Table 3:** Compounds determined by LC-MS/MS in apple juice and ciders fermented using single-culture and triple mixed-cultures.

Number	Compounds	AJ	Treatment (*n* = 3) (μg/L)					
			SCA	SPL1	SPL2	SPL3	SPL4	SPL5
	Organic acids							
1	Acrylic acid	2452.89 ± 81.19 b	594.13 ± 19.72 c	2627.98 ± 17.83 a	37.94 ± 12.95 d	2726.85 ± 86.45 a	27.8 ± 1.39 d	52.85 ± 3.99 d
2	Glyoxylic acid	22.56 ± 3.6 d	35.68 ± 2.28 b	30.80 ± 1.99 c	34.92 ± 1.34 b	34.36 ± 0.92 bc	43.53 ± 2.72 a	41.55 ± 1.32 a
3	Propionic acid	7.52 ± 0.31 c	8.79 ± 0.39 b	8.80 ± 0.25 b	8.61 ± 0.35 bc	10.14 ± 1.19 a	8.66 ± 0.52 bc	9.10 ± 0.92 ab
4	Glycolic acid	194.05 ± 2.14 b	108.56 ± 3.86 c	247.80 ± 7.53 a	36.83 ± 5.27 d	231.17 ± 19.00 a	48.42 ± 13.94 d	37.11 ± 7.82 d
5	Malonic acid	107.72 ± 50.95 c	162.42 ± 19.81 abc	126.88 ± 15.82 bc	194.2 ± 48.33 a	122.76 ± 4.63 bc	175.78 ± 29.08 ab	214.11 ± 9.44 a
6	Palmitic acid	289.12 ± 37.20 c	833.94 ± 18.51 b	1076.68 ± 148.63 a	966.30 ± 54.73 a	997.63 ± 54.39 a	1061.71 ± 47.81 a	1062 ± 57.34 a
7	Pyruvic acid	90.77 ± 8.57 cd	75.53 ± 2.5 d	118.19 ± 2.37 b	83.09 ± 6.75 d	136.67 ± 22.85 a	92.24 ± 8.21 cd	103.46 ± 6.08 ab
8	Itaconic acid	124.84 ± 34.45 f	545.67 ± 24.07 c	1347.57 ± 21.21 a	480.59 ± 8.00 d	1046.01 ± 48.14 b	187.49 ± 20.5 e	109.21 ± 11.97 f
9	L-malic acid	2390.08 ± 74.17 c	1556.56 ± 42.16 c	1645.72 ± 24.01 c	1245.80 ± 20.04 a	965.81 ± 21.81 b	998.79 ± 22.93 b	1081.30 ± 19.02 ab
10	Maleic acid	36.22 ± 4.19 e	97.50 ± 7.99 d	133.17 ± 4.33 c	106.03 ± 4.90 d	101.61 ± 8.44 d	183.24 ± 6.68 b	194.64 ± 3.67 a
11	(R)-3-hydroxybutyric acid	2.95 ± 0.14 f	72.88 ± 7.47 b	97.10 ± 3.56 a	49.55 ± 2.02 c	22.07 ± 0.97 e	75.14 ± 2.78 b	32.27 ± 1.28 d
12	Quinic acid	12,757.83 ± 74.6 d	16,465.97 ± 52.83 b	15,222.81 ± 81.15 c	16,609.83 ± 74.89 b	35,706.86 ±82.50 a	16,225.68 ± 65.5 b	16,861.02 ± 74.85 b
13	Maslinic acid	3.47 ± 0.28 d	11.09 ± 1.2 a	5.61 ± 0.13 c	7.86 ± 1.7 b	2.83 ± 0.15 d	8.31 ± 0.42 b	10.69 ± 2.17 a
14	Malic acid	116.65 ± 1.15 e	153.26 ± 4.69 c	167.31 ± 2.17 b	134.23 ± 3.29 a	141.98 ± 13.83 cd	123.11 ± 16.36 de	115.50 ± 2.73 a
15	Succinic acid	29.45 ± 0.40 e	9591.50 ± 20.43 b	958.75 ± 49.66 d	7086.79 ± 55.05 c	878.43 ± 35.84 d	9600.65 ± 47.45 b	10,141.63 ± 40.74 a
16	Fumaric acid	71.71 ± 7.32 b	130.39 ± 9.21 ab	87.04 ± 6.46 ab	118.64 ± 38.84 ab	86.82 ± 2.39 ab	128.39 ± 46.2 ab	136.17 ± 51.68 a
17	Phenoxyacetic acid	2.03 ± 1.37 d	8.33 ± 0.76 b	11.82 ± 1.71 a	7.73 ± 0.18 b	10.72 ± 0.34 a	7.27 ± 0.64 b	4.73 ± 0.75 c
18	cis-aconitic acid	7.25 ± 1.65 e	904.89 ± 22.74 b	944.47 ± 112.67 b	592.57 ± 7.49 d	1064 ± 3.07 a	745.83 ± 20.46 c	1031.97 ± 18.18 a
19	Citric acid	1069.28 ± 109.11 f	5039.46 ± 26.10 d	2407.29 ± 142.65 e	8242.77 ± 382.28 b	6583.95 ± 333.00 c	9564.43 ± 171.39 a	2343.84 ± 47.19 e
20	2-isopropylmalic acid	35.64 ± 11.78 e	2447.36 ± 104.28 c	1698.46 ± 20.58 d	3945.29 ± 4.50 b	2797.50 ± 150.74 c	17,209.54 ± 698.97 a	17,272.17 ± 607.54 a
21	Succinic acid semialdehyde	57.14 ± 4.33 e	307.24 ± 17.9 b	215.11 ± 2.99 c	357.86 ± 2.84 a	169.26 ± 11.65 d	342.41 ± 10.21 a	344.73 ± 13.45 a
22	Hydroxyphenyllactic acid	96.77 ± 13.82 f	499.86 ± 15.11 a	184.73 ± 4.22 e	343.44 ± 9.07 d	179.13 ± 3.48 e	383.38 ± 14.76 c	424.30 ± 4.22 b
23	D-glucuronic acid	48.60 ± 8.21 f	6072.45 ± 21.05 b	4604.97 ± 63.66 d	4130.15 ± 282.25 e	5731.96 ± 152.62 c	4154.09 ± 184.11 e	8264.68 ± 112.71 a
24	Phenyllactic acid	3.37 ± 1.91 f	6109.55 ± 116.28 a	1937.18 ± 66.41 d	3659.06 ± 90.28 c	1235.05 ± 61.84 e	5449.24 ± 253.25 b	6149.70 ± 79.66 a
25	Trans-ferulic acid	-	36.00 ± 1.22 b	37.39 ± 1.68 b	25.62 ± 3.51 c	46.53 ± 5.11 a	29.10 ± 5.16 c	45.62 ± 0.29 a
26	Isocitric acid	460.32 ± 17.58 e	15,829.76 ± 795.11 a	3268.20 ± 217.93 bc	2814.06 ± 308.02 c	3460.85 ± 183.61 b	1364.85 ± 131.83 d	1697.86 ± 54 d
27	D-tartaric acid	8.60 ± 0.48 c	7.40 ± 0.44 c	14.50 ± 3.82 a	9.84 ± 1.56 bc	13.29 ± 1.35 a	9.53 ± 0.81 bc	11.93 ± 1.16 ab
28	Shikimic acid	181.52 ± 41.81 b	101.94 ± 3.63 cde	80.10 ± 0.91 e	235.12 ± 11.52 a	96.21 ± 7.57 de	131.42 ± 5.24 c	120.91 ± 3.42 cd
29	Trans-aconitic acid	2.33 ± 0.8 d	81.48 ± 28.29 ab	51.58 ± 3.36 c	50.49 ± 7.93 c	99.40 ± 12.31 a	68.33 ± 5.57 bc	75.60 ± 3.43 b
30	(R)-mandelic Acid	-	29.87 ± 0.39 a	23.02 ± 0.28 cd	24.32 ± 0.21 c	22.50 ± 1.63 cd	21.53 ± 1.57 d	27.07 ± 2.89 b
31	Phenylpyruvic acid	-	274.21 ± 31.92 d	542.66 ± 23.74 a	162.60 ± 5.53 e	455.96 ± 16.02 b	354.34 ± 29.06 c	380.55 ± 15.56 c
32	Mesaconic acid	196.14 ± 81.4 g	4594.37 ± 193.92 ab	1662.10 ± 97.65 e	4408.71 ± 76.38 bc	1192.82 ± 55.93 f	4728.14 ± 194.18 a	4266.08 ± 67.07 d
33	4-hydroxyphenylpyruvic acid	-	1702.39 ± 105.92 b	141.08 ± 29.12 e	796.59 ± 36.39 d	11.54 ± 13.27 f	1410.06 ± 111.92 c	2598.33 ± 39.59 a
34	L-lactic acid	59.15 ± 6.88 e	64.92 ± 3.82 e	181.82 ± 0.66 cd	192.47 ± 1.69 bc	193.86 ± 3.85 b	176.93 ± 3.47 d	282.63 ± 14.26 a
35	Acetic acid	1081.96 ± 55.07 b	1412.84 ± 23.46 a	1036.56 ± 22.19 b	1436.59 ± 31.6 a	1365.09 ± 54.61 a	885.11 ± 17.57 c	749.22 ± 24.4 d
36	Terephthalic acid	7.07 ± 5.38 f	208.48 ± 14.43 b	153.48 ± 7.78 d	242.22 ± 9.15 a	81.09 ± 5.09 e	153.39 ± 2.93 d	191.68 ± 1.48 c
37	3-hydroxymethylglutaric acid	515.91 ± 88.33 d	1073.30 ± 43.42 a	1133.88 ± 77.81 a	764.04 ± 81.28 bc	1212.90 ± 69.74 a	625.19 ± 99.14 cd	906.52 ± 97.37 b
38	Kojic acid	15.30 ± 1.41 c	21.55 ± 1.07 bc	20.38 ± 2.47 bc	28.82 ± 0.73 a	24.99 ± 3.58 ab	21.14 ± 0.36 bc	22.62 ± 0.12 bc
39	Vanillic acid	10.82 ± 1.23 e	81.58 ± 4.07 b	59.13 ± 5.46 d	73.20 ± 3.45 c	56.11 ± 4.47 d	53.82 ± 1.85 d	94.62 ± 2.15 a
40	Syringic acid	5.65 ± 1.03 d	17.24 ± 3.57 ab	8.37 ± 0.64 cd	19.13 ± 1.38 a	13.03 ± 1.5 bc	16.75 ± 1.23 ab	21.24 ± 3.63 a
41	Phenylacetic acid	4.29 ± 0.85 f	15.29 ± 1.69 c	9.81 ± 0.22 d	25.72 ± 0.81 a	7.61 ± 0.43 e	17.32 ± 1.1 b	18.27 ± 0.96 b
42	Abscisic acid	13.81 ± 3.75 b	78.78 ± 4.64 a	88.64 ± 3.46 a	69.71 ± 5.12 a	75.39 ± 3.29 a	107.18 ± 58.88 a	75.95 ± 1.35 a
43	Vanillylmandelic acid	-	195.93 ± 8.74 a	120.80 ± 5.14 b	148.29 ± 5.25 b	85.52 ± 5.96 c	145.57 ± 23.01 b	143.20 ± 4.21 b
44	Phthalic acid	25.47 ± 8.38 ab	-	38.15 ± 1.27 a	14.86 ± 1.05 bc	27.19 ± 7.05 ab	5.54 ± 1.14 c	-
	Ʃ_(Sum)_	22,606.26 ± 582.12 e	77,660.35 ± 1280.71 a	44,577.86 ± 1331.79 d	60,034.50 ± 1151.67 c	69,533.42 ± 730.11 b	77,163.20 ± 2078.21 a	77,774.61 ± 1299.05 a
	Polyphenols							
1	o-cresol	0.43 ± 0.01 e	17.22 ± 1.73 d	13.70 ± 2.47 d	55.82 ± 4.78 c	2.38 ± 0.32 e	127.05 ± 6.37 b	227.73 ± 11.97 a
2	p-cresol	0.21 ± 0.07 c	4.76 ± 0.28 b	5.02 ± 0.12 b	5.37 ± 0.55 b	5.05 ± 0.68 b	5.10 ± 0.52 b	6.40 ± 0.53 a
3	Epicatechin	21.25 ± 1.33 d	76.10 ± 2.93 c	94.51 ± 3.80 a	77.68 ± 6.77 c	98.16 ± 6.68 a	71.04 ± 0.45 c	85.67 ± 0.76 b
4	Caffeic acid	-	821.44 ± 6.2 c	941.15 ± 14.74 b	1044.59 ± 21.54 a	864.57 ± 30.87 d	927.12 ± 12.23 b	823.04 ± 14.61 c
5	Catechin	169.10 ± 18.49 d	2544.85 ± 76.94 b	1641.13 ± 49.78 c	4008.82 ± 69.16 a	3112.65 ± 71.84 d	3765.71 ± 86.35 a	3698.22 ± 46.84 a
6	m-coumaric acid	-	1310.58 ± 189.10 e	3038.27 ± 25.43 a	1430.39 ± 40.67 f	2782.09 ± 108.46 b	1726.14 ± 128.25 d	2053.44 ± 53.24 c
7	Chlorogenic acid	537.46 ± 12.59 a	82.34 ± 15.79 d	121.78 ± 20.42 c	45.26 ± 4.15 e	219.81 ± 6.04 b	22.76 ± 2.15 f	30.88 ± 1.09 ef
8	Genistein	14.61 ± 2.59 d	52.84 ± 5.9 b	38.15 ± 0.49 c	66.08 ± 3.58 a	32.89 ± 2.52 c	48.95 ± 4.34 b	60.35 ± 4.51 a
9	4-methylcatechol	4.38 ± 0.71 bc	6.44 ± 0.99 a	3.70 ± 0.2 c	5.76 ± 0.80 a	5.71 ± 0.56 a	5.17 ± 0.24 ab	5.56 ± 0.73 ab
10	Quercetin	1.32 ± 0.34 c	100.68 ± 12.64 b	299.10 ± 30.62 a	72.34 ± 2.76 bc	245.53 ± 18.79 a	36.71 ± 13.16 bc	96.16 ± 11.23 b
11	Mulberrin	90.78 ± 2.42 d	547.25 ± 37.55 b	618.91 ± 9.26 a	559.70 ± 15.14 b	475.85 ± 25.38 c	644.57 ± 30.57 a	567.41 ± 19.57 b
12	Quercitrin	18.20 ± 1.20 c	219.87 ± 45.48 ab	312.12 ± 43.23 a	199.94 ± 7.11 ab	269.43 ± 16.99 ab	127.54 ± 28.72 bc	150.33 ± 6.61 abc
13	Quercetin 3-galactoside	3.64 ± 0.23 b	28.16 ± 15.72 ab	42.95 ± 7.88 a	13.58 ± 2.01 b	42.36 ± 27.15 a	6.74 ± 2.89 b	23.02 ± 19.03 ab
14	Procyanidin B2	19.95 ± 3.53 f	56.92 ± 3.41 c	146.48 ± 5.24 b	33.95 ± 3.25 e	156.10 ± 2.13 a	30.70 ± 2.92 e	41.17 ± 1.64 d
15	Limocitrin	0.02 ± 0.00 e	33.56 ± 1.05 c	1.77 ± 0.48 e	25.83 ± 0.83 d	1.75 ± 0.19 e	49.24 ± 7.98 a	42.20 ± 4.61 b
16	Phloretin	225.22 ± 26.54 c	832.38 ± 66.18 b	947.22 ± 26.44 b	1183.27 ± 131.96 a	1072.22 ± 34.75 a	949.39 ± 33.71 b	1084.25 ± 59.76 a
17	(-)-epigallocatechin	0.97 ± 0.61 f	17.40 ± 1.4 a	11.68 ± 0.53 bc	12.55 ± 0.49 b	10.21 ± 1.34 cd	6.95 ± 0.82 e	9.44 ± 0.66 d
18	Phlorizin	1092.96 ± 117 e	4126.56 ± 43.78 d	4579.12 ± 362.24 bc	5568.79 ± 76.08 a	4798.19 ± 63.58 b	4419.42 ± 89.94 cd	4884.58 ± 80.77 b
19	Naringenin	-	78.52 ± 1.06 b	154.74 ± 5.45 a	190.69 ± 15.13 a	121.58 ± 5.98 ab	157.44 ± 6.43 a	155.97 ± 4.6 a
20	Sinapic acid	2.41 ± 0.12 d	10.82 ± 0.58 c	10.60 ± 0.78 c	12.75 ± 0.31 ab	14.24 ± 1.06 a	12.16 ± 1.14 bc	12.16 ± 1.71 bc
21	Myricetin	0.52 ± 0.22 d	11.87 ± 2.67 b	5.68 ± 1.15 c	10.99 ± 1.57 b	4.75 ± 1.01 cd	15.32 ± 2.5 ab	18.62 ± 2.75 a
22	Bergapten	6036.42 ± 236.66 a	4708.60 ± 402.07 a	4663.11 ± 195.31 a	4923.59 ± 115.54 b	5838.48 ± 78.85 a	4817.93 ± 168.40 b	4735.09 ± 199.71 b
23	Quercetin 3-arabinoside	4.17 ± 0.20 e	20.24 ± 1.52 b	27.40 ± 2.41 a	11.77 ± 0.82 cd	25.15 ± 3.57 a	9.27 ± 0.70 d	13.65 ± 1.73 c
24	3-hydroxybenzoic acid	3.60 ± 1.08 e	9.75 ± 0.93 b	16.49 ± 0.83 a	11.85 ± 1.06 cd	7.04 ± 0.5 a	10.29 ± 1.34 d	11.94 ± 0.30 c
25	Daidzin	2.27 ± 0.19 d	11.02 ± 1.42 ab	11.78 ± 1.03 b	5.67 ± 0.38 c	12.44 ± 1.23 a	6.76 ± 0.31 c	9.66 ± 0.83 b
26	Gallic acid	1.24 ± 0.17 f	156.67 ± 5.39 d	237.25 ± 25.09 bc	324.78 ± 17.39 a	124.47 ± 4.19 e	245.71 ± 15.63 b	214.70 ± 7.38 c
27	2-benzylbutanedioic acid	43.93 ± 2.67 a	18.72 ± 1.52 d	37.32 ± 0.80 b	17.43 ± 2.94 d	45.60 ± 0.81 a	15.13 ± 1.41 d	24.29 ± 1.74 c
28	Glabranin	1.99 ± 0.11 d	7.51 ± 0.86 c	10.98 ± 0.31 a	11.89 ± 1.02 a	9.23 ± 0.97 b	11.85 ± 1.12 a	12.46 ± 1.14 a
29	Isoquercitrin	0.56 ± 0.02 d	10.73 ± 0.69 b	6.7 ± 0.18 c	14.74 ± 2.85 a	2.59 ± 0.37 d	6.80 ± 0.97 c	5.93 ± 0.28 c
30	Curcumin	16.92 ± 3.07 d	121.32 ± 5.44 a	27.08 ± 0.44 c	108.39 ± 2.06 b	26.52 ± 2.53 c	111.04 ± 6.72 b	104.56 ± 7.72 b
31	Psoralen	1.66 ± 0.48 d	62.26 ± 15.37 a	56.08 ± 3.31 a	39.40 ± 1.00 b	63.33 ± 2.18 a	22.29 ± 1.67 c	43.89 ± 2.69 b
32	Hesperidin	2.15 ± 0.26 b	0.91 ± 0.2 d	1.53 ± 0.33 c	1.17 ± 0.09 cd	3.34 ± 0.37 a	-	-
33	Naringin	2.16 ± 0.06 bc	1.76 ± 0.63 cd	2.83 ± 0.70 b	2.51 ± 0.35 bc	1.26 ± 0.06 de	0.59 ± 0.04 e	4.63 ± 0.03 a
34	4-aminophenol	3.72 ± 0.22 c	5.49 ± 0.14 a	3.90 ± 0.54 c	5.29 ± 0.32 ab	4.70 ± 0.48 b	4.67 ± 0.32 b	5.38 ± 0.33 a
	Ʃ_(Sum)_	8324.24 ± 2239.42 c	16,115.55 ± 721.92 b	18,130.21 ± 1630.51 ab	20,102.62 ± 336.23 ab	20,616.35 ± 684.21 a	20,984.22 ± 5239.72 a	19,296.12 ± 797.07 ab
	Terpenoids							
1	Geranylacetate	25.76 ± 0.86 c	26.88 ± 3.98 c	396.22 ± 71.64 b	396.71 ± 45.90 b	51.49 ± 4.58 c	41.09 ± 0.55 c	694.00 ± 53.14 a
2	Bornyl acetate	1.47 ± 0.04 d	35.94 ± 8.98 cd	73.45 ± 3.84 c	15.39 ± 4.09 d	655.98 ± 58.16 a	14.28 ± 1.00 d	251.55 ± 28.97 b
3	Myrcene	1.63 ± 0.02 e	66.25 ± 2.92 b	74.02 ± 2.64 a	41.59 ± 2.05 c	25.79 ± 4.03 d	36.91 ± 2.54 c	74.81 ± 3.33 a
4	Nerylacetate	21.32 ± 1.79 f	65.19 ± 2.83 e	266.40 ± 28.79 b	175.01 ± 1.22 c	751.54 ± 14.82 a	146.69 ± 21.69 d	187.85 ± 0.11 c
5	(+)-camphor	27.46 ± 0.71 f	27.63 ± 2.42 f	361.25 ± 21.34 a	83.12 ± 4.61 d	60.04 ± 6.89 e	150.39 ± 9.21 c	168.39 ± 7.31 b
6	Terpinine-4-ol	2.80 ± 0.88 d	2.60 ± 0.32 d	16.72 ± 0.60 b	21.82 ± 2.01 b	82.90 ± 7.74 a	9.03 ± 0.72 c	5.14 ± 0.81 cd
7	Citronellyl acetate	2.69 ± 0.25 e	5.80 ± 0.10 e	27.48 ± 6.67 d	227.53 ± 22.14 a	14.22 ± 0.40 de	113.75 ± 8.76 b	56.50 ± 10.70 c
8	Alpha-terpineol; Patulin	28.24 ± 5.52 a	1.41 ± 0.08 e	14.06 ± 1.98 c	25.18 ± 0.96 a	19.55 ± 4.14 b	2.82 ± 0.41 de	6.62 ± 0.21 d
	Ʃ_(Sum)_	111.39 ± 7.48 g	231.69 ± 15.56 f	1229.59 ± 57.48 c	986.35 ± 60.28 d	1661.52 ± 48.05 a	514.96 ± 11.86 e	1444.85 ± 59.73 b
	Ʃ _(Sum: LC-MS)_	31,041.89 ± 1697.44 e	94,007.59 ± 1229.36 ab	63,837.66 ± 1737 d	81,089.47 ± 729.40 c	91,735.63 ± 510.01 b	98,665.72 ± 6781.31 a	98,515.58 ± 465.02 a

Note: “-” means not detected. Data with different letters (a, b, c, d, e, f, g) within each row are significantly different (*p* < 0.05).

## Data Availability

The data presented in this study are available on request from the corresponding author.
